# Development of a Clioquinol Nanocarrier as a New, Promising Option for the Treatment of Dermatomycosis

**DOI:** 10.3390/pharmaceutics15020531

**Published:** 2023-02-04

**Authors:** Simone Jacobus Berlitz, Paula Reginatto, Gabriella da Rosa Monte Machado, Alexandre Meneghello Fuentefria, Fernando Dal Pont Morisso, Renata Vidor Contri, Irene Clemes Külkamp-Guerreiro

**Affiliations:** 1 Programa de Pós-Graduação em Nanotecnologia Farmacêutica, Laboratório de Pesquisa em Tecnologia Farmacêutica e Cosmética Aplicada, Faculdade de Farmácia, Universidade Federal do Rio Grande do Sul, Av. Ipiranga, 2752, Santana, Porto Alegre 90610-000, Brazil; 2 Programa de Pós-Graduação em Microbiologia Agrícola e do Ambiente, Universidade Federal do Rio Grande do Sul, Rua Sarmento Leite, 500, Santana, Porto Alegre 90050-170, Brazil; 3 Programa de Pós-Graduação em Ciências Farmacêuticas, Universidade Federal do Rio Grande do Sul, Av. Ipiranga, 2752, Santana, Porto Alegre 90610-000, Brazil; 4 Programa de Pós-Graduação em Tecnologia de Materiais e Processos Industriais, Universidade Feevale, RS-239, 2755, Vila Nova, Novo Hamburgo 93525-075, Brazil

**Keywords:** cutaneous diseases, clioquinol, nanotechnology, antifungal, dermatomycosis

## Abstract

Dermatomycosis is a common fungal infection, and its treatment is limited by few antifungal agents. Clioquinol (CQ) is an antiparasitic agent that has been studied for new uses, such as antifungal and antiviral applications. CQ was incorporated into a lipid-based nanocarrier as a new, promising option for dermatomycosis. This study aimed to develop a CQ-loaded lipid-based nanocarrier for cutaneous application and to evaluate its antifungal activity. CQ-loaded nanoformulation (LBN-CQ) was developed using the ultrasonication method, and the particle size, polydispersity index (PDI), pH, zeta potential, and drug content were monitored for 45 days. To evaluate antifungal activity, broth microdilution and a time-kill assay were performed. LBN-CQ presented a particle size of 91 ± 3 nm and PDI of 0.102 ± 0.009. The zeta potential and pH values were −9.7 ± 2.0 mV and 6.0 ± 0.1, respectively. The drug content was 96.4 ± 2.3%, and the encapsulation efficiency was 98.4%. LBN-CQ was able to reduce the minimum inhibitory concentration (MIC) in a 2-fold or 4-fold manner in most of the tested strains. Additionally, LBN-CQ presented stable fungistatic action that was not concentration- or time-dependent. In conclusion, the developed CQ-loaded nanocarrier is a promising treatment for skin fungal infections and a promising candidate for future randomized clinical trials.

## 1. Introduction

Dermatomycosis is considered one of the most commonly occurring infective diseases. Its treatment is usually limited by few antifungal agents that can present side effects, and resistance to those drugs can appear [1,2,3]. These infections are mainly caused by dermatophytic filamentous fungi and yeast, such as *Candida* spp. [4,5]. Fungal infections are a major health concern, especially in immunocompromised patients [6,7]. Therefore, there is a need for new antifungal agents, and the repositioning of old drugs to treat fungal infections could be a promising option due to its advantages, such as low drug development costs and time [8,9,10]. In this context, the drug clioquinol (CQ) could be an old drug candidate for treating dermatomycosis.

**Figure 1:**
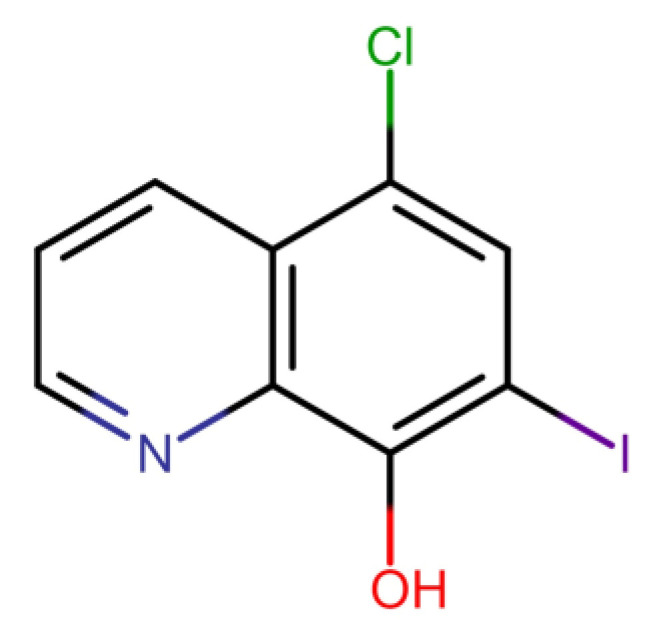
Chemical structure of clioquinol (CQ).

Clioquinol (CQ, 5-chloro-7-iodo-8-quinolinol) is an 8-hydroxyquinoline derivative that was used as antiparasitic agent during the decades of 1950, 1960, and 1970 [8]. CQ was withdrawn from the market in the ’70s due to an association to subacute myelo-optic neuropathy (SMON) however this association is circumstantial and was never validated [8,9,10]. According to the World Health Organization, CQ is classified as antiprotozoal [11]. Besides its antiparasitic properties, CQ has been studied regarding its chelating, anticancer, antifungal, and antiviral properties, with potential uses in diseases such as Alzheimer and Parkinson’s, as well as COVID-19 [8,9,10,12,13,14,15]. Several research groups are studying CQ, CQ complexes, and 8-hidroxyquinolines derivatives and their application as antifungal agents [9,16,17,18,19,20,21,22,23,24]. CQ presents an iodine in the 7-position and a chlorine in the 5-position of the quinoline ring, which may be related to its antifungal activity (Figure 1) [9].

Several old drugs are not in use anymore considering that they were not able to be incorporated in conventional formulations or dosage forms due to issues such as poor bioavailability and physical-chemical instability [25]. The development of advanced topical carriers can overcome these challenges as well as propose new antifungal formulations that are in need due to fungal resistance of conventional drugs [25,26]. Considering this scenario, the use of lipid-based nanocarriers has great potential because of their ability to overcome drug resistance, decrease systemic toxicity, and target the site of infection [25]. Loading a drug in a nanocarrier is an alternative to improve its solubility, as well as to provide a controlled release that could increase drug residence time in the skin [27,28,29]. Furthermore, loading antifungal agents in nanocarriers usually presents superior efficacy with decreased toxicity [26,30,31]. In this context, the use of nanotechnology in topical formulations can bring more advantages since it allows a topical therapy of the infection site with minimal drug absorption or systemic side effects [29,32,33]. Since CQ is highly lipophilic and it is not soluble in water, its incorporation in nanostructured systems is interesting and has been described previously [12,34,35,36,37]. However, these studies did not evaluate the use of CQ as an antifungal agent but focused on its use as an option for the treatment of neurodegenerative diseases [12,34,35,36,37]. Moreover, no study exploring lipid-based nanocarriers to load CQ were published to date. Lipid-based nanosystems are promising for topical formulations due to their fluidic nature and surfactant interface that allow an uniform spreading of the product in the skin making drug penetration easier while maintaining high biocompatibility with the lipid composition of the skin [33,38,39,40]. Considering the advantages of lipid nanocarriers, monoolein was the chosen lipid for this work. Monoolein is a biocompatible, non-toxic and biodegradable lipid mainly used for drug delivery applications since it can incorporate several types of molecules for different applications, including the formation of nanocarriers [41,42,43,44].

Hence, finding a new therapeutical use for an already approved drug is an interesting approach. The use of nanotechnology to incorporate drugs can aid in broadening the possibilities of drug repurposing since it can overcome biopharmaceutical challenges such as low aqueous solubility. CQ has been studied as a new antifungal agent but the development of new pharmaceutical formulations aiming this activity is rare. Therefore, loading CQ in a lipid-based nanocarrier is a promising strategy to aid the development of pharmaceutical formulations aiming antifungal efficiency against strains that cause dermatomycosis.

## 2. Materials and Methods

### 2.1. Preparation of CQ-Loaded Nanocarrier

The nanocarriers were prepared using a probe sonicator (QR550W, Eco-Sonics, Indaiatuba, SP, Brazil). Primarily, in a glass beaker, CQ (12.5 mg) was dissolved in DMSO (500 µL). Then, poloxamer 407 (1250 mg), monoolein (2500 mg), and ultrapure water (20 mL) were added to that beaker. The mixture in the beaker was sonicated using the probe sonicator for 20 min at 99% amplitude for the formation of the nanocarriers. The nanocarrier solution was allowed to cool at room temperature and then was transferred to a 25 mL volumetric flask. The final volume was adjusted to 25 mL with ultrapure water. Theorical CQ concentration of the nanocarrier is 0.5 mg/mL.

### 2.2. Nanocarrier Characterization

Mean hydrodynamic size and polydispersity index (PDI) were determined by dynamic light scattering (DLS) at an angle of 90° in previously diluted samples with ultrapure water (1:500, *v*/*v*) (NanoBrook 90Plus PALS, Brookhaven Instruments, Holtsville, NY, USA) [29,45]. The same equipment was used to measure zeta potential of previously diluted samples (1:1000, 1 mM KCl) by electrophoretic mobility [29,45].

The pH value was measured by potentiometry (NanoBrook 90Plus PALS, Brookhaven Instruments, USA). A previously calibrated (pH 4.0 and pH 7.0) electrode probe was placed directly in the samples.

All measurements were carried out in triplicate.

### 2.3. Drug Content

A UV/Vis spectrophotometric method was developed to determine CQ content using a LAMBDA 265 UV/Vis spectrophotometer (Perkin Elmer, Waltham, MA, USA) connected to UV LAB 4.0 software. The method was validated regarding the parameters of linearity, precision, and accuracy as per guidelines by International Conference on Harmonization (ICH) [46]. The absorbance of CQ samples was measured at 254 nm using 1 cm quartz cell. A stock solution of 1 mg/mL concentration was prepared by dissolving CQ and 50 µL of a placebo nanoformulation in methanol. From the stock solution, standard solutions of 2.0, 3.0, 4.0, 5.0, 6.0, 7.0, and 8.0 µg/mL were prepared by diluting with methanol. The linear response for that concentration range of CQ was recorded with the coefficient of determination of 0.9953.

Intraday and interday precision were found to be 3.29% and 3.45%, respectively, which are within the acceptable limits. To evaluate CQ amount in the nanoformulation, a 5µg/mL solution of LBN-CQ in methanol was prepared, vortexed for 2 min and had its absorbance read. To evaluate encapsulation efficiency, ultrafiltration-centrifugation method was used, using a filter device (Amicon^®^ 10000 MW, Millipore, Burlington, MA, USA) [29,47]. Centrifugation was performed at 4000 rpm for 30 min (Eppendorf 5417R) and drug content in the ultrafiltrate was determined by UV/Vis. The encapsulation efficiency (EE%) was calculated according to Equation (1).

Encapsulation efficiency (EE%) = (total drug content − free drug content)/(total drug content) × 100 (1)

### 2.4. Fourier Transform Infrared Spectroscopy (FT-IR)

Fourier transform infrared spectroscopy (FT-IR) was carried out using a Perkin Elmer UATR (Perkin Elmer, USA) spectrophotometer equipped with attenuated total reflectance (ATR) accessory [34]. A resolution of 4 cm^−1^ from 4000 to 400 cm^−1^ was used for LBN-CQ, LBN-BL and a CQ solution. The background spectrum of air was scanned under the same instrumental conditions before each series of measurements. Sixteen scans were taken of the background and the samples to obtain an average spectrum.

### 2.5. Thermal Analysis

The nanoformulations LBN-CQ, LBN-BL, each component of the nanosystem, and their physical mixture in the same proportions used in LBN-CQ were assessed using thermogravimetry analysis (TGA) [34]. The TGA analysis was performed using a TGA-51H (Shimadzu, Kyoto, Japan) device, under nitrogen atmosphere at a heating rate of 10 °C per minute within a temperature range of 20 °C to 700 °C.

### 2.6. Fungal Strains

A total of nine fungal strains were tested [48]. Among them, five were *Candida* strains: *Candida albicans* (CA MT07), *C. parapsilosis* (CP MT12), *C. glabrata* (CG MT04), *C. tropicalis* (ATCC 750), and *C. krusei* (ATCC 6258). The other four were dermatophyte strains: *Microsporum canis* (MCA HCPA 12), *M. gypseum* (MGY50), *Trichophyton rubrum* (ATCC 28188), and *T. mentagrophytes* (TME15). *Candida* strains were analyzed phenotypically by Vitek Yeast Biochemical Card (BioMerieux Vitek, Hazelwood, MO, USA). All strains are from the mycology collection of yeast of Federal University of Rio Grande do Sul (Porto Alegre, Rio Grande do Sul, Brazil). Standard strains (ATCC 750, 6258 and 28188) were obtained from American Type Culture Collection (ATCC; Manassas, VA, USA).

### 2.7. In Vitro Antifungal Susceptibility Testing

The in vitro susceptibility test to determine the mininum inhibitory concentrations (MICs) of the samples was performed using the broth microdilution method [49,50]. For *Candida* strains, the assay was performed according to protocol M27-A3, and for dermatophytic species, the M38-A2 protocol was used [49,50].

Briefly, in a 96-well microplate, fungal strains were inoculated in RPMI 1640 medium in the presence of different concentrations of the antifungal agent. Then, the microplates were incubated for 48 h (*Candida* spp.) or 96 h (dermatophytic strains), and after this time, the fungal growth was assessed. MICs were defined as the lowest concentration of the substance at which the tested microorganism did not demonstrate visible growth. The assays were conducted in duplicate, in three independent assays. Concentrations ranging from 0.24 to 250 µg/mL of the LBN-CQ were evaluated, as well as LBN-BL. CQ in solution (DMSO, 2%, *v*/*v*) was used as control.

### 2.8. Time Kill Assay

Nanoformulations containing CQ at MIC, MIC×2, MIC×4, and MIC×8 concentrations were prepared in RPMI 1640 medium and evaluated against *Candida krusei* (ATCC 6528) and a dermatophytic strain (*Trichophyton rubrum*—ATCC 28188), according to previous works [51,52]. The fungal inoculum suspension of each strain was added to nanoformulation to obtain a concentration of approximately 1–5 × 10^5^ and 1–5 × 10^3^ CFU/mL for yeast and for dermatophyte, respectively. A solution containing only fungal inoculum in RPMI 1640 medium was used as a growth control. In parallel, an unloaded nanoformulation was also used. All formulations and controls were prepared in triplicate. The determination of time-kill kinetics curves was performed at 0, 24, and 48 h for yeasts, and 0, 24, 48, and 96 h for dermatophytes. Then, 20 μL of each sample were plated on Sabouraud Dextrose Agar using a Drigalski spreader. The plates were incubated at 35 °C for 48 h for yeast, and 32 °C for 96 h for dermatophyte. A fungistatic or fungicidal action was defined when a decrease <99.9% log_10_ CFU/mL or ≥99.9% log_10_ CFU/mL was observed, respectively. This effect is compared to the starting inoculum at time zero [51,52].

### 2.9. Sorbitol Protection Assay

Integrity of the fungal cell wall was evaluated using sorbitol protection assay [53]. MICs of LBN-CQ, LBN-BL, and CQ in solution were determined by broth microdilution method according to protocols M27-A3 and M38-A2 for *Candida* spp. and dermatophytes, respectively [49,50]. The MICs were determined in the absence and presence of 0.8 M sorbitol added to the RPMI 1640 growth medium as an osmoprotectant. Micafungin was used as positive control. MICs were measured for 7 days [53].

### 2.10. Statistical Analysis

The software GraphPad Prism^®^ 7.02 (San Diego, CA, USA) was used for statistical analysis. The results were analyzed for statistical significance by analysis of variance (ANOVA) followed by a post-hoc test for multiple comparisons (Tukey’s test) or *t*-test when comparing two groups, both with a significance level of 0.05.

## 3. Results and Discussion

### 3.1. Nanostructure Characterization

The nanostructure hydrodynamic size of the drug loaded nanocarrier (LBN-CQ) was 91 ± 3 nm with a PDI of 0.102 ± 0.009, indicating a monomodal size distribution. The zeta potential was −9.7 ± 2.0 mV, suggesting that the stability of the nanosystem is mainly due to a steric effect [54]. The pH of the nanoformulation was 6.0 ± 0.1, which is suitable for skin application [55]. The drug content was 96.4 ± 2.3% (*w*/*v*), and the encapsulation efficiency (EE%) was 98.4%. Several factors can influence the encapsulation efficiency, including the type of nanocarrier and its components, as well as the drug characteristics [25,29]. Due to the lipidic nature of the developed nanocarrier and the lipophilicity of clioquinol, a high encapsulation efficiency is expected, and it also suggests that the chosen components are suitable for loading this specific drug [12,29,34]. A blank lipid-based nanocarrier (LBN-BL), without the drug, was also evaluated and achieved hydrodynamic size of 95 ± 2 nm with a PDI of 0.166 ± 0.001, a zeta potential of −11.7 ± 3.3 mV, and a pH of 5.74 ± 0.6. There was no difference between the drug-loaded and blank nanocarrier parameters except for the PDI. In the LBN-BL, PDI was higher but also indicated a monomodal size distribution since PDI values in the range of 0.1 to 0.25 suggests a narrow size distribution [56,57]. Other studies loaded CQ in nanocarriers, but they explored only polymeric nanocarriers [34,35,36]. A high encapsulation efficiency (97%) was also achieved in a study that loaded CQ in poly(methyl methacrylate-co-acrylic acid (P(MMA-co-AA)) nanoparticles [34]. A lower encapsulation efficiency of 50 to 60% was achieved using polymeric n-butyl-2-cyanoacrylate (PBCA) nanoparticles [35]. Regarding particle size, both studies achieved smaller particle sizes that ranged from 50 to 100 nm [34,35]. In another study, CQ was loaded in human serum albumin nanoparticles, and these nanocarriers presented a drug-loading efficiency of 41% and a particle size of around 13 to 15 nm [36]. CQ was also incorporated in polymeric micelles, but no characterization of the system was performed, and no particle size in the nanoscale was described [37].

**Figure 2:**
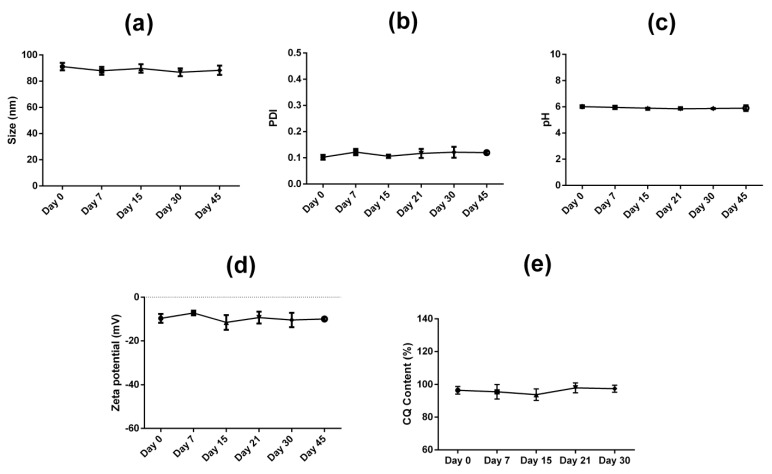
Characterization of the nanocarrier containing clioquinol (LBN-CQ): mean particle size (**a**), polydispersity index (PDI) (**b**), pH (**c**), zeta potential (**d**) and clioquinol content (**e**). All samples were stored at room temperature for 45 days.

The parameters of hydrodynamic size, PDI, pH, zeta potential, and CQ content from the drug-loaded nanocarrier (LBN-CQ) were monitored for 45 days (Figure 2). All the evaluated parameters were considered stable during that time interval, with no statistical differences.

**Figure 3:**
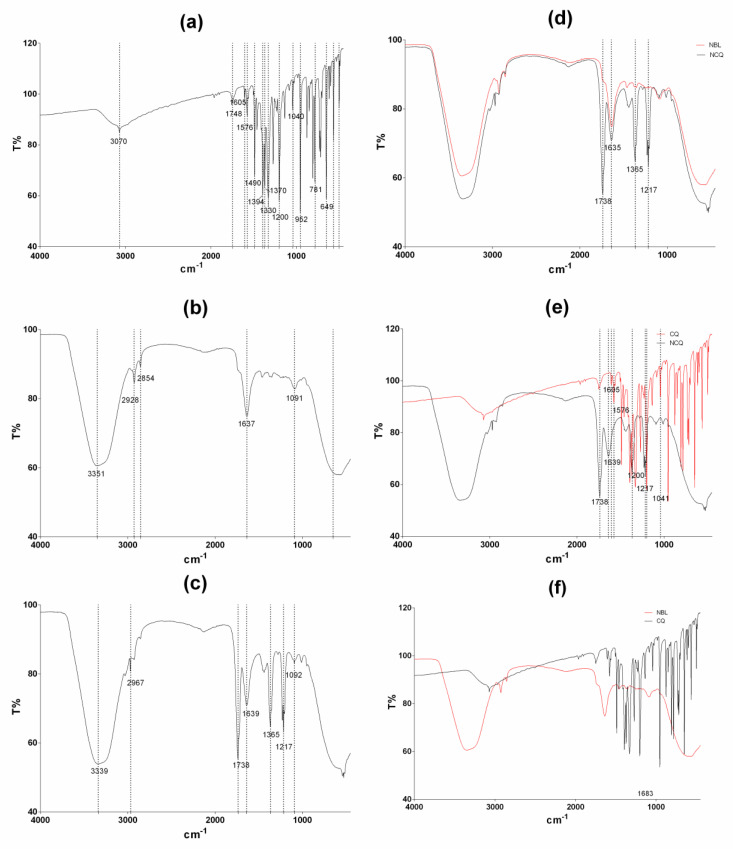
FT-IR of bulk CQ (**a**), a placebo nanocarrier (**b**), CQ-loaded nanocarrier (LBN-CQ) (**c**), the spectra of placebo and LBN-CQ (**d**), the spectra of bulk CQ and LBN-CQ (**e**) and the spectra of bulk CQ and LBN-BL (**f**).

The FT-IR spectroscopy is one of the most used techniques to assess the interactions of compounds [58,59]. The FT-IR spectra of LBN-BL, LBN-CQ, and CQ in solution are shown in Figure 3a–c, respectively. Some characteristic peaks of CQ were found at 3070 cm^−1^ (ν(O-H) + ν(C-H)), 1605 cm^−1^ (ν(C=N)), 1576 cm^−1^ (ν(C=C)), 1490 cm^−1^ (ν(CC)), 1200 cm^−1^ (ν(CO)), and 1040 cm^−1^ (C-Cl) (Figure 3a) [60,61]. Regarding LBN-BL and LBN-CQ spectra, most bands appeared in the same wavelength range (Figure 2d). However, the peaks observed at 1738 cm^−1^, 1365 cm^−1^, and 1217 cm^−1^ can be attributed to the presence of CQ in LBN-CQ. Comparing the spectra of CQ and LBN-CQ (Figure 3e), the disappearance of characteristic bands of CQ at 1576 cm^−1^ and 1041 cm^−1^ and band shifts from 1605 cm^−1^ to 1639 cm^−1^ and from 1200 cm^−1^ to 1217 cm^−1^ were observed. The disappearance or shifts of characteristic bands from CQ can indicate interactions of the drug with the nanosystem, reiterating its presence in the nanocarrier [62,63,64,65,66]. These findings, along with the high encapsulation efficiency, could be an indication of drug encapsulation in the nanoformulation [62,63,64,65,66].

**Figure 4:**
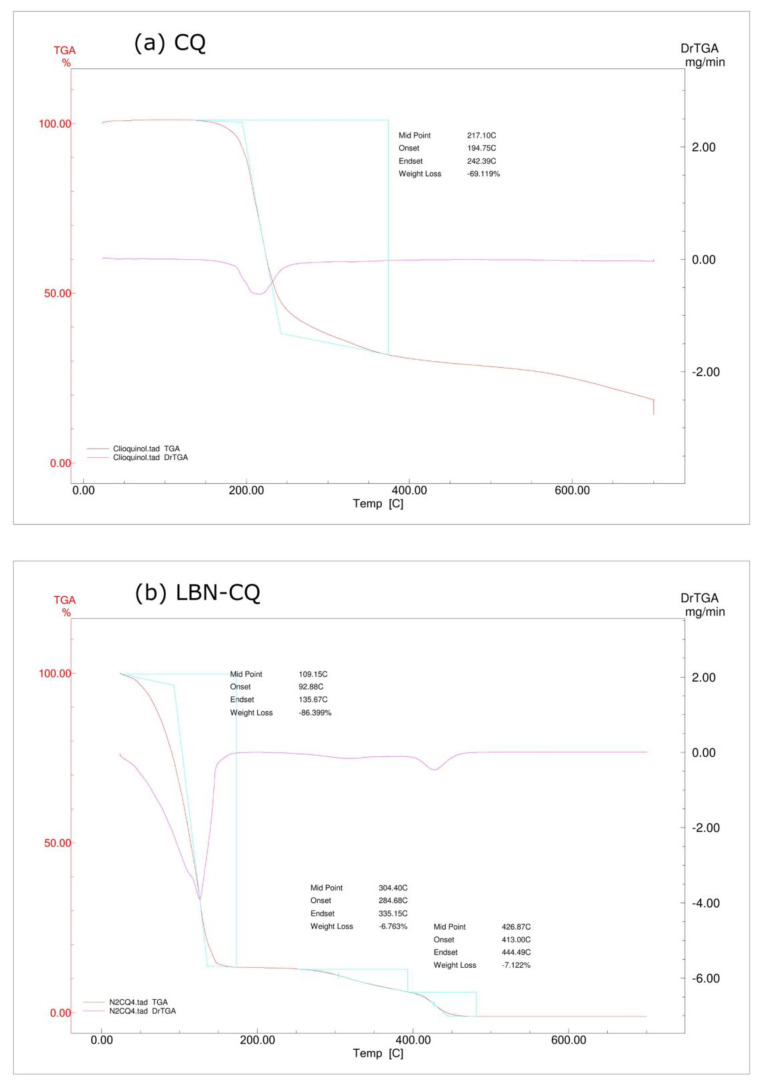
Thermogravimetric analysis thermogram of CQ (**a**) and LBN-CQ (**b**).

TGA analyses of CQ and LBN-CQ are presented in Figure 4. The TGA curves show that CQ was thermally stable up to 194.75 °C and that its decomposition ended at 242.39 °C, while the mass loss was 69.12%. Considering LBN-CQ, three decomposition steps were observed. A first mass loss of 86.40% occurred between 92.88 °C and 135.67 °C and could be attributed partially to a loss of water from the system. The second step started at 284.68 °C and finished at 335.15 °C with a mass loss of 6.76%. The third step comprises a mass loss of 7.122% in the range of 413.00 °C and 444.49 °C. These results suggests that the method of production and the temperature used do not appear to degrade the constituents of the nanoformulation.

### 3.2. In Vitro Antifungal Susceptibility Testing

Table 1 presents the MICs for LBN-CQ, LBN-BL and CQ in solution. The nanocarrier LBN-CQ was more effective (MIC range −0.24–0.97 µg/mL) against tested fungal strains when compared to the MIC values obtained from a solution of clioquinol (MIC range −0.5–4.0 µg/mL). The nanoformulation was able to achieve a 4-fold decrease in MIC in most of the strains tested (ATCC 750, ATCC 6258, MCA HCPA 12, MYG50). Only the MIC values of TME15 and CA MT07 were not significantly altered by the encapsulation of CQ in the nanocarrier. When the fungal strains were evaluated against LBN-BL, there was no growth inhibition, indicating that the nanoformulation components do not have any antifungal effect.

The improvement of the antifungal action for LBN-CQ was observed against both yeasts and dermatophytes since the growth of most of the strains was strongly inhibited by LBN-CQ. This may be related to the improved solubility of CQ in the nanoformulation.

Other studies evaluated MIC for CQ and the compound showed antifungal activity for dermatophytes, *Candida* spp. and even for *Fusarium* species [9,16,18]. Although previous publications evaluated the antifungal activity of CQ, none of them developed a drug delivery system.

Even though CQ appears to have higher MIC than terbinafine, its use in association with other antifungal drugs such as terbinafine is promising, as it could allow a lower dosage use and also increase the action spectrum [16].

### 3.3. Time Kill Assay

**Figure 5:**
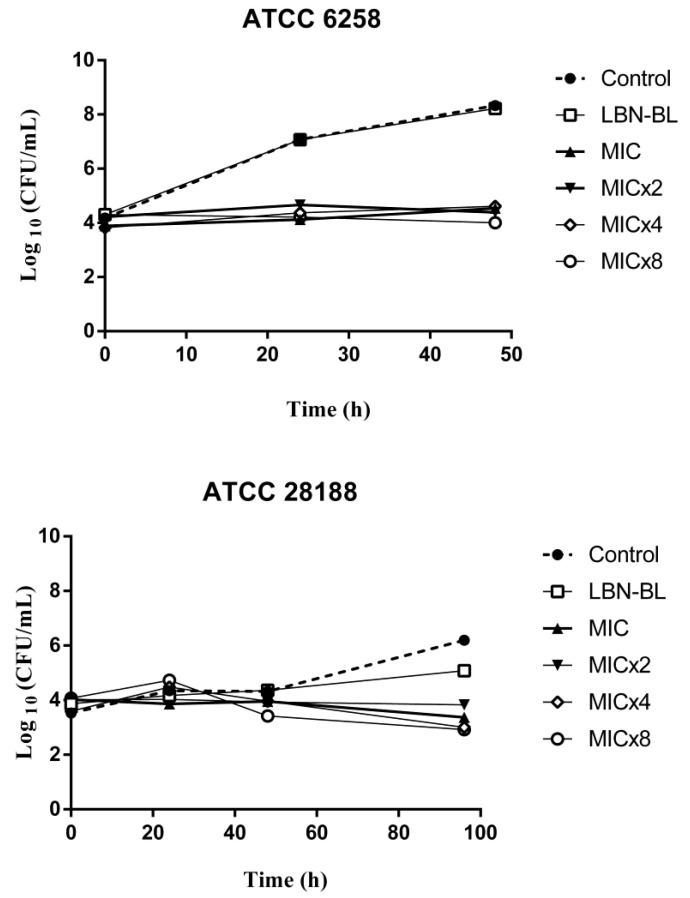
Log plots of killing kinetics against *C. krusei* (ATCC 6258) and *T. rubrum* (ATCC 28188) for the concentrations of the nanoformulation containing clioquinol (LBN-CQ) at MIC×8, MIC×4, MIC×2, MIC, the control and the placebo nanoformulation (LBN-BL).

As described, LBN-CQ was able to maintain antifungal efficacy (Figure 5). The nanoformulation presented a similar behavior for all test concentrations (MIC, MIC×2, MIC×4, MIC×8), where a decrease in the growth of *C. albicans* was observed. For *T. rubrum*, a decrease of CFU/mL was perceptible for all concentrations starting from 48 h.

Hence, the evaluation of the dose-response kinetic curves demonstrates that LBN-CQ presents a stable fungistatic action, capable of reducing both yeast and dermatophytic growth for up to 48 and 96 h at all concentrations evaluated (MIC, MIC×2, MIC×4, MIC×8) when compared to the untreated control. Thus, the fungistatic action was not dependent on concentration or time tested.

A fungistatic effect of CQ was also observed for *M. canis* (MCA01) and *C. albicans* (ATCC 18804), but a fungicidal effect was observed for *T. mentagrophytes* (TME40) [9]. On the other hand, You et al. reported that CQ exhibited a fungicidal effect that was time- and concentration-dependent for *C. albicans* (SC5314) [21]. A fungicidal effect of CQ was also observed for *T. rubrum* (TRU47) [16]. All these studies used different strains, which may partly explain the various results.

As expected, LBN-BL behaved similarly to the untreated control, being unable to inhibit fungal growth.

### 3.4. Sorbitol Protection Assay

Fungi with defective cell walls are unable to grow at normal conditions [67]. However, fungal growth can be achieved even with a defective cell wall if the growth medium is supplemented with an osmotic protectant such as sorbitol [68]. In this condition, if the antifungal agent acts on fungal cell wall, MIC values will increase [4,21,53]. Therefore, a sorbitol protection assay was employed to verify if LBN-CQ could affect the integrity of the fungal cell wall. The MIC values, after incubation with sorbitol, increased in a 2-fold manner for *C. albicans* (CA MT07), *C. parapsilosis* (CP MT12), *C. tropicalis* (ATCC 750), *M. canis* (MCA HCPA 12), *Trichophyton rubrum* (ATCC 28188), and *T. mentagrophytes* (TME15) (Table 1). For *C. glabrata* (CG MT04), MIC values increased in a 4-fold manner, while for *C. krusei* (ATCC 6258), the MIC remained the same (Table 1). 

The increase of the MIC values when the medium was supplemented with sorbitol indicates that the nanoformulation was able to maintain the ability of CQ to target fungal cell wall for most of the tested strains, as reported by previous study that evaluated the mechanism of action of CQ in several dermatophyte and *Candida* spp. strains [53]. However, the MIC value of *C. krusei* ATCC 6258 remained the same. It has been reported that CQ did not damage the cell wall directly on *C. albicans* SC5314 and ATCC 10231 strains [21]. The divergency regarding these results could be due to the different strains used.

CQ is a promising antifungal agent, and its status as an already approved drug shortens the development time and costs of new pharmaceutical formulations. Moreover, new, rapidly available antifungal options are needed, considering the rise of fungal drug resistance [25,26,40]. Additionally, the treatments of dermatomycosis are often long and require prolonged use of drugs, compromising patient compliance [26]. Hence, the use of novel drug delivery systems, such as the lipid-based nanocarrier proposed in this work, can overcome these problems since LBN-CQ presented lower MIC values when compared to unencapsulated drug and, therefore, could lead to an enhanced clinical efficacy and reduce dosing frequency [26].

CQ has been shown to be neurotoxic when used orally, so its incorporation in a topical nanocarrier is desired and a safer option to avoid systemic toxicity [10]. Pharmaceutical nanosystems containing antifungal agents usually decrease drug systemic toxicity while targeting the site of the infection, so the use of more expensive drug delivery systems could also be justified [25,26]. However, clinical trials in humans should be performed in future works. Even though the employment of new technologies such as nanocarriers can lead to higher production costs, the need for a lower drug dose that nanosystems allows could balance these costs. In addition, the ultrasonication technology explored in this work for the production of LBN-CQ is easily scaled up and does not use organic solvents [69].

## 4. Conclusions

In the present investigation, a nanocarrier containing CQ (LBN-CQ) was produced with a non-ionic surfactant and an amphiphilic lipid with high encapsulation efficiency. The physicochemical characteristics of this system were within the nanoscale and, along with drug content, were constant for at least 45 days. Furthermore, LBN-CQ was able to increase antifungal activity when compared to both the unloaded nanocarrier and CQ in solution. In addition, LBN-CQ showed fungistatic action, while LBN-BL was not able to inhibit fungal growth. Therefore, the present investigation presents an innovative formulation that used nanotechnology as a strategy to repurpose CQ and to allow cutaneous application, demonstrating the potential use of this nanocarrier in the treatment of dermatomycosis. However, clinical trials in humans must be assessed in the future to confirm the efficacy of the nanoformulation on the clinical treatment of dermatomycosis.

## Figures and Tables

**Table 1:** Minimum inhibitory concentration (MIC) values at µg/mL for LBN-CQ, LBN-BL and CQ in solution against yeasts and dermatophytes.

Strains/Agents	LBN-CQ (µg/mL)		CQ (µg/mL)	LBN-BL
		MIC Increase after Addition of Sorbitol		
ATCC 750	0.24	*	1.0	-
ATCC 6258	0.24	=	1.0	-
CA MT07	0.48	**	0.5	-
CG MT04	0.48	****	1.0	-
CP MT12	0.48	**	1.0	-
MCA HCPA 12	0.97	**	4.0	-
MGY50	0.48	**	2.0	-
TME15	0.97	**	1.0	-
ATCC 28188	0.48	**	1.0	-

*Candida tropicalis* (ATCC 750), *C. krusei* (ATCC 6258), *C. albicans* (CA MT07), *C. glabrata* (CG MT04), *C. parapsilosis* (CP MT12); *Microsporum canis* (MCA HCPA 12), *M. gypseum* (MGY50), *Trichophyton mentagrophytes* (TME15), and *T. rubrum* (ATCC 28188). Nanoformulation containing clioquinol at 64 µg/mL: LBN-CQ, unloaded nanoformulation: LBN-BL; CQ: clioquinol in solution at 64 µg/mL. (-): fungal growth due to lack of antifungal efficacy of the unloaded nanoformulation. * indicates 1-fold increase, ** indicates a 2-fold increase; **** indicates a 4-fold increase; = indicates no MIC difference.

## Data Availability

The data presented in this study are available on request from the corresponding author.
